# Artificial Intelligence and Terrestrial Point Clouds for Forest Monitoring

**DOI:** 10.1007/s40725-024-00234-4

**Published:** 2024-12-27

**Authors:** Maksymilian Kulicki, Carlos Cabo, Tomasz Trzciński, Janusz Będkowski, Krzysztof Stereńczak

**Affiliations:** 1 IDEAS NCBR, ul. Chmielna 69, 00–801 Warsaw, Poland; 2 Institute of Fundamental Technological Research, Polish Academy of Science, ul. Pawińskiego 5B, 02–106 Warsaw, Poland; 3 Department of Mining Exploitation and Prospecting, University of Oviedo, Campus de Mieres, 33600 Mieres, Oviedo, Spain; 4 Warsaw University of Technology, pl. Politechniki 1, 00–661, Warsaw, Poland; 5 Tooploox, ul. Tęczowa 7, 53–601, Wrocław, Poland; 6 Department of Geomatics, Forest Research Institute, ul. Braci Leśnej 3, 05–090 Sękocin Stary, Poland

**Keywords:** Deep learning, Machine learning, Forest inventory, Tree characteristics, Open data, Precision forestry, LiDAR, TLS

## Abstract

**Purpose of Review:**

This paper provides an overview of integrating artificial intelligence (AI), particularly deep learning (DL), with ground-based LiDAR point clouds for forest monitoring. It identifies trends, highlights advancements, and discusses future directions for AI-supported forest monitoring.

**Recent Findings:**

Recent studies indicate that DL models significantly outperform traditional machine learning methods in forest inventory tasks using terrestrial LiDAR data. Key advancements have been made in areas such as semantic segmentation, which involves labeling points corresponding to different vegetation structures (e.g., leaves, branches, stems), individual tree segmentation, and species classification. Main challenges include a lack of standardized evaluation metrics, limited code and data sharing, and reproducibility issues. A critical issue is the need for extensive reference data, which hinders the development and evaluation of robust AI models. Solutions such as the creation of large-scale benchmark datasets and the use of synthetic data generation are proposed to address these challenges. Promising AI paradigms like Graph Neural Networks, semi-supervised learning, self-supervised learning, and generative modeling have shown potential but are not yet fully explored in forestry applications.

**Summary:**

The review underscores the transformative role of AI, particularly DL, in enhancing the accuracy and efficiency of forest monitoring using ground-based 3D point clouds. To advance the field, there is a critical need for comprehensive benchmark datasets, open-access policies for data and code, and the exploration of novel DL architectures and learning paradigms. These steps are essential for improving research reproducibility, facilitating comparative studies, and unlocking new insights into forest management and conservation.

## Introduction

LiDAR (Light Detection and Ranging) is a remote sensing method that uses light pulses to create detailed 3D representations of an environment. In the past, forestry research relied primarily on aerial LiDAR, which provided a comprehensive view of forests from above the canopy. However, interest in ground-based LiDAR technology is growing. These scanners operate at close range below the tree canopy, allowing for very detailed mapping of the tree stems, branches and undergrowth that is not possible with aerial solutions.

Ground-based scanners were initially limited to static Terrestrial Laser Scanners (TLS), but the technology has expanded to Mobile Laser Scanners (MLS), including Personal Laser Scanners (PLS) carried by humans. These devices allow for continuous data collection while moving around the area, which speeds up data collection. They come in various forms, such as handheld devices, in backpacks or mounted on vehicles. LiDAR scanners have become cheaper and more available in recent years, making it easier than ever to capture detailed 3D data. The data captured with LiDAR takes the form of a point cloud, an unordered collection of points in 3D space. As a rule, each point is labelled with an intensity value that can potentially provide information about the physical properties of the scanned surface.

Precision forestry represents a paradigm in forest management and conservation that utilizes technology to obtain information about forests at the individual tree level. Ground-based LiDAR scanners play a crucial role in data collection for precision forestry, enabling the mapping of tree stems and branches with an unprecedented level of detail. With the advent of such detailed data collection comes a critical need for appropriate data processing techniques. In this context, the distinction between rule-based (heuristic) approaches and Artificial Intelligence (AI) methodologies, particularly Machine Learning (ML) and Deep Learning (DL), becomes pivotal.

Rule-based systems operate on predefined sets of instructions or algorithms developed from expert knowledge. While these systems offer predictability and transparency, their efficacy is constrained by the complexity of the rules and the variability of natural environments, which may not be fully encapsulated in static rules. On the other hand, AI, through ML and DL, marks a significant advancement in data processing for precision forestry. ML, the development of algorithms that learn and make predictions based on data, and DL, a subset of ML that uses multi-layer neural networks to learn highly complex data representations, are well-suited for handling the volume and complexity of LiDAR data. Unlike heuristic methods, AI does not rely on predefined rules but learns directly from data, enabling it to adapt to new, unseen data and improve over time. This adaptability makes AI an invaluable tool in the modern forester’s arsenal, capable of efficiently processing large datasets and uncovering insights that heuristic approaches might overlook.

### Overview of AI Methods for Point Cloud Processing

**Fig. 1:**
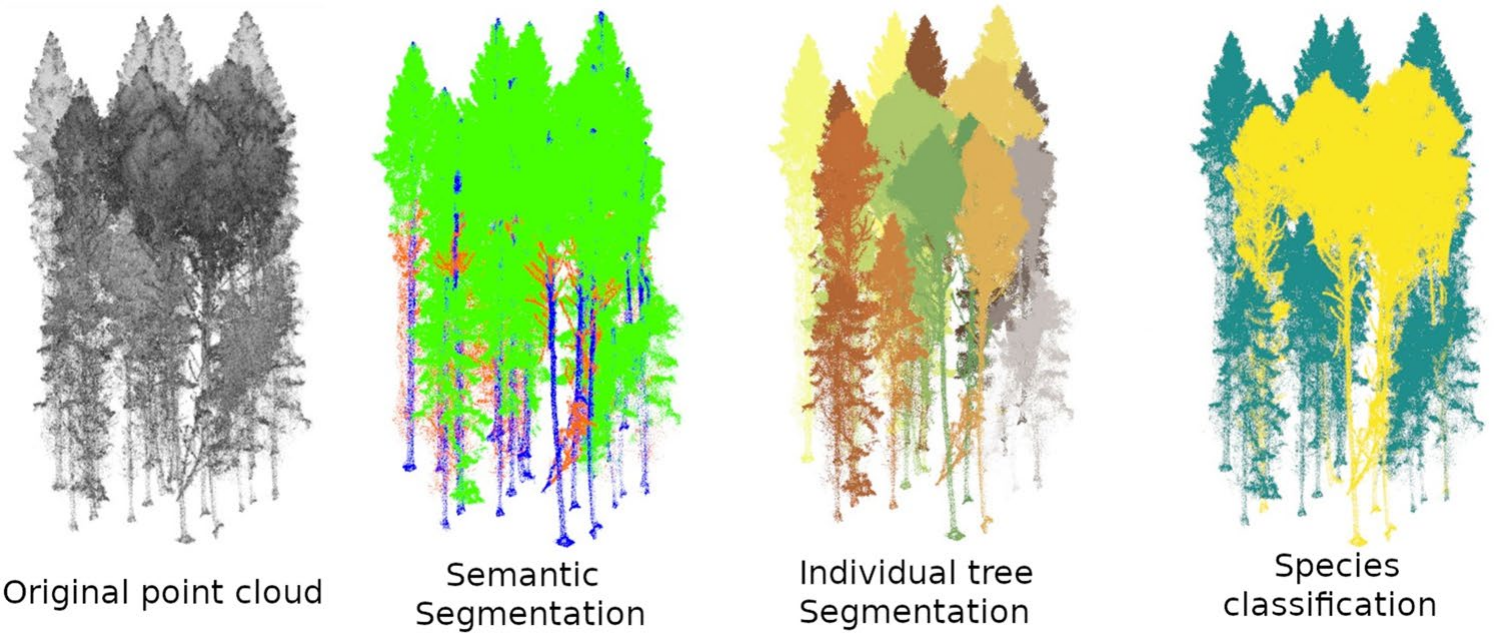
A visualization of various AI tasks and corresponding information that can be extracted from forest point clouds

In the context of point cloud data, AI models can perform a variety of processing tasks that provide useful insights about 3D environments. Three key tasks, visualized in Fig. 1, are particularly relevant to forestry applications:

- Point Cloud Classification: This task involves assigning a single label to an entire point cloud, based on its overall characteristics. The model learns to map the input point cloud to a predefined set of categories. In forestry, this can be applied to identify tree species or to assess their health status.
- Semantic Segmentation: This process assigns a semantic label to each individual point in the cloud, partitioning the point cloud into semantically meaningful regions. In forest environments, this can be used to distinguish between different types of vegetation, such as separating leaves, branches, and trunks.
- Instance Segmentation: Related to semantic segmentation, this task divides semantic categories into distinct individual object instances. In forestry, this is crucial for delineating individual trees within a larger forest point cloud, enabling tree-level analysis and inventory.

AI techniques can be also employed in point cloud reconstruction and completion, where missing parts of the cloud are inferred, and in regression tasks, which predict continuous variables based on point cloud data. Although these latter tasks were not present in the reviewed articles, they are significant areas of research in the broader field of point cloud analysis and could perhaps find their application in forest research.

Before we delve into the details of the reviewed papers, we provide a broad overview of the types of AI models present in the field.

### Machine Learning Based on Handcrafted Features

Machine learning encompasses a wide range of techniques that enable computers to learn from data and make predictions without being explicitly programmed. Traditionally, ML models have relied heavily on feature engineering, which can be defined as the creation of descriptive features designed by domain experts to capture the relevant information and reduce the data complexity. There are many classes of models that can be trained on such features. Common examples include Random Forest (RF) [1], Support Vector Machine (SVM) [2], Multi-layer Perceptron (MLP) [3] or XGBoost [4]. Compared to more complex deep neural networks, these traditional ML models are much faster to compute and can successfully learn from small datasets. However, their performance relies heavily on the selection of features. Below we go over various kinds of features used for forest data.

### Point Geometric Features

A popular approach to extract features of individual points, proposed by Hackel et al. [5], involves geometric descriptions of the neighbourhood of a point, defined based on a fixed distance or the nearest k neighbors. Based on the locations of points in the neighbourhood, a covariance matrix *C* is derived in Eq. 1:

1 $$C\left({N}_{R}\right)=\frac{1}{N}\sum_{p\in N}{\left(p-p\right)\left(p-p\right)}^{T}$$

The eigenvectors **e**_1_*,* **e**_2_*,* **e**_3_ ∈ R^3^ and eigenvalues λ_1_ ⩾ *λ*_2_ ⩾ *λ*_3_ ∈ R of this covariance matrix are used to calculate various features providing information about the shape of the neighborhood. Table 1 show definitions of features from Hackel et al. [5].

**Table 1:**
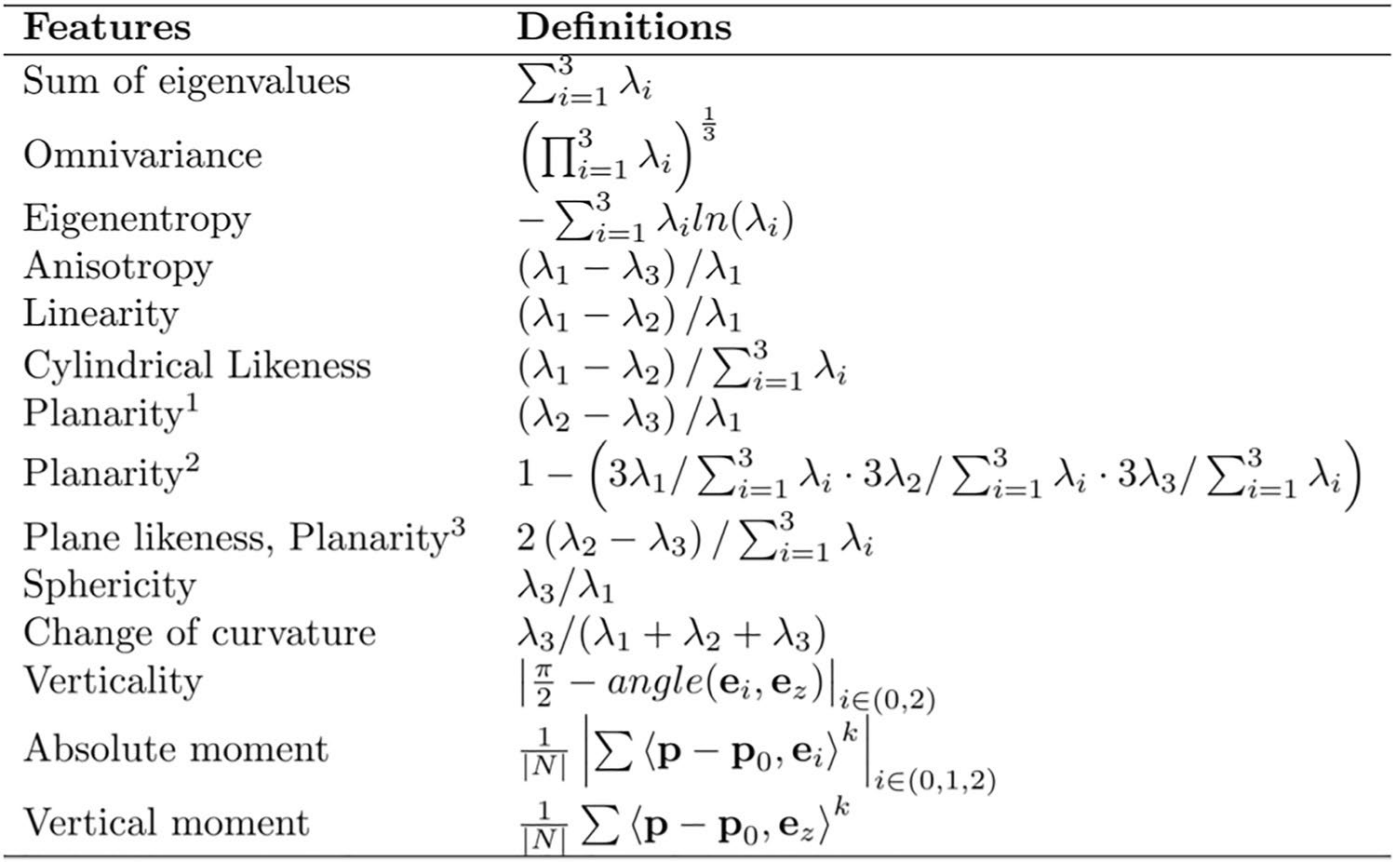
Local neighbourhood features of points and their definitions [ 5 ].

Some approaches [6–8] include several neighbourhood scales and calculate the features separately for each scale.

### Tree-Level Features

When dealing with problems on single-tree level, such as species classification, one needs tree-level features. A variety of attributes can be extracted from the point cloud at tree level, including:

- properties of the whole point cloud, such as its height, total number of points, volume of the convex hull [9, 10]
- aggregated properties of the points, e.g. mean intensity, median height, or geometric features [11, 12]
- Hui et al. [10] propose fractal features, derived by counting the voxels at different levels of point cloud voxelization

A unique method of describing tree structure involves converting the point cloud into a Qualitative Structure Model (QSM) [13], which represents stems and branches as a hierarchical set of cylinders, approximating their actual shape. A QSM model, among other use cases, can be used to provide features for ML models, such as average branch angle, length, volume [14, 15]. Since the QSM contains information about the branching structure, features can be considered separately at different branch hierarchy levels.

### Deep Learning

While processing point clouds using handcrafted features offers interpretability and efficiency, Deep Learning (DL) has recently emerged as a powerful alternative. Unlike traditional ML that relies on handcrafted features, DL models derive the features directly from the data. The complexity of these models means that they typically require large datasets to perform well.

**Fig. 2:**
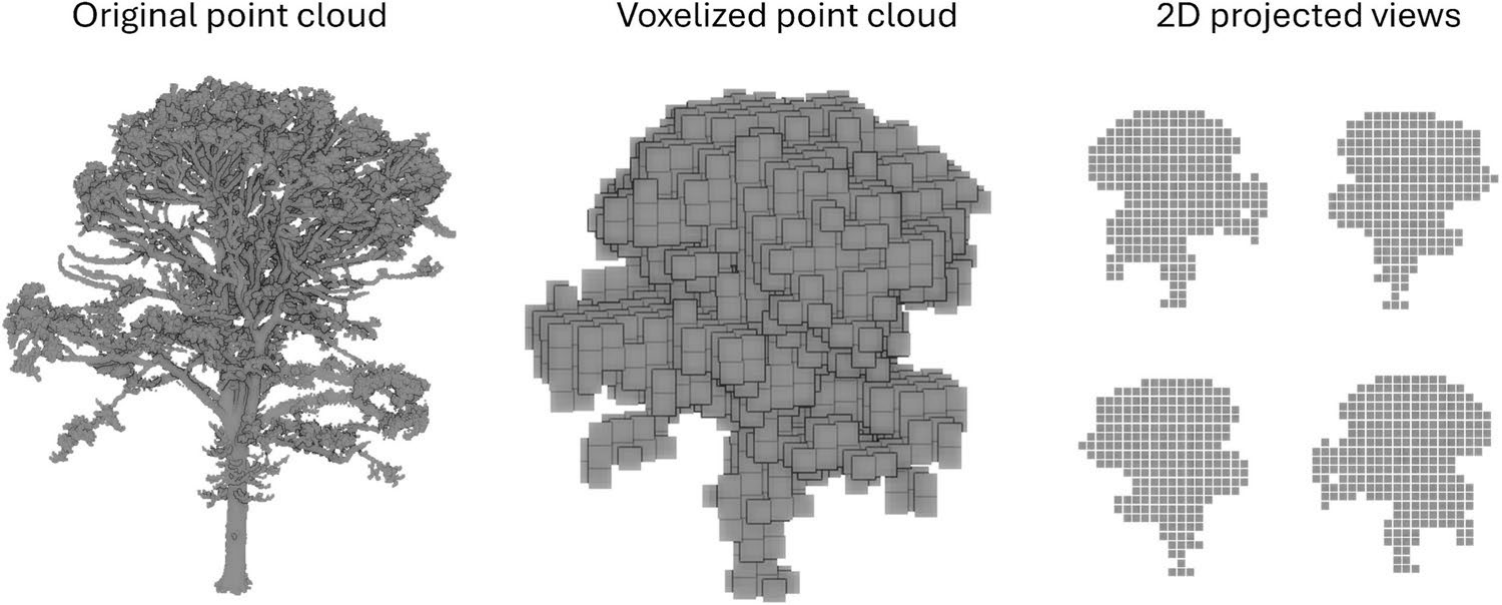
Different methods of representing a tree point cloud. Left: raw, unprocessed point cloud. Middle: Point cloud represented as a 3D grid of voxels. Right: point cloud projected as 2D images viewed from various angles

Dealing with point cloud data has been challenging for DL models for a variety of reasons. Firstly, point clouds are inherently unordered, and all processing has to be order-invariant—given the same set of points in a different order, the output must be the same. This makes point cloud data fundamentally different from domains such as text, which is a 1-dimensional sequence of letters, or images, which form a well-structured 2D grid. In addition, point clouds often have uneven point density and vary in terms of points per sample. To address these challenges, several approaches have been developed. Many of them involve converting the point cloud to other formats, as visualized in Fig. 2.

### Convolutional Neural Networks

Convolutional Neural Networks (CNNs) have been extraordinarily successful in processing grid-structured data, with CNN architectures such as YOLO[16] and ResNet [17] establishing themselves as a standard approach for image analysis. CNNs work by applying learnable filters across the input data, capturing local patterns and hierarchical features. While initially designed for 2D image data, CNNs can be naturally extended to 3D grids, making them applicable to point cloud data after preprocessing.

**Projection-based methods:** The 2D projection approach involves projecting 3D point clouds onto 2D planes, typically incorporating depth information. A variant of this method, popular in forestry, is the Canopy Height Model (CHM), which is a top-down 2D projection of the canopy. While some methods are based on a single projected image [18], most of them integrate views from various angles. Typically, features extracted from different images are aggregated and jointly processed by a classification model [19].

**Voxel-based methods:** Voxel conversion discretizes 3D space into a grid of voxels (3D pixels) and assigns voxel attributes based on point data. This can be done with simple binary occupancy, indicating the presence of any points in a voxel, or with more complex representations such as point density or aggregated point attributes. Some CNN architectures have been developed specifically to process 3D voxels, with VoxNet [20] being an early example. Additionally, many image processing CNNs, such as VGG[21] or ResNet, have been successfully adapted to 3D data. However, adding another dimension significantly increases the need for computational resources.

Computational requirements of 3D convolution can be reduced using the fact that voxel grids derived from point clouds tend to be rather sparse, with a large proportion of empty voxels. This enables the utilization of specialized deep learning architectures that can efficiently handle sparse data, such as MinkowskiNet [22].

### Point-Based Methods

While DL on point clouds remains relatively undeveloped compared to other modalities, there is a growing number of methods in the area.

PointNet, a seminal work introduced by Qi et al. [23], is the first prominent DL model to operate directly on point clouds. It has shown remarkable performance in both classification and segmentation tasks, setting a precedent in the field. Building upon PointNet, PointNet +  + was developed by the same authors [24], addressing its inability to capture local structures. PointNet +  + introduced a hierarchical structure that applies PointNet recursively on nested partitions of the point set. It demonstrated significant improvements over its predecessor, particularly in tasks requiring detailed local understanding.

Multiple recent works succeeded at adapting established architectures from other domains in a way that is compatible with point clouds. One such adaptation involves the use of convolutions, with notable approaches including PointCNN [25] and PointConv [26]. Another approach, PointMixer [27], works by adapting the MLP-Mixer architecture, originally designed for image processing.

### Graph Neural Networks

Graph Neural Networks (GNNs) have shown promise in processing point cloud data by representing the point cloud as a graph, where each point is a node and edges represent relationships between points. This approach allows the model to capture local geometric structures and global context simultaneously. GNNs have been effectively applied in various domains such as social network analysis, molecular chemistry, and recommendation systems. Notable architecture types include Graph Convolutional Networks (GCNs), which apply convolutions to graph data, and Graph Attention Networks (GATs), which leverage attention mechanisms to weigh the importance of neighboring nodes.

A popular graph architecture for processing point clouds is Dynamic Graph CNN (DGCNN) [28]. It works by constructing a neighborhood graph from the point cloud and processes it using graph convolutions. The graph is dynamically updated in each layer, allowing the model to capture different levels of local structure. DGCNN is often used as an alternative to PointNet and fits a similar niche.

A notable application of GNNs in forestry is the work by Chattoraj et al.[29], which proposes a species recognition framework using an ARMA (AutoRegressive Moving Average) GNN architecture.

Despite their potential, the use of GNNs in forestry applications remains limited, likely due to the complexity of implementation and the need for large, well-structured datasets.

### Transformers

Transformer models, which have now become the new state of the art in natural language processing and computer vision tasks, have also been adapted for point cloud processing. These models use self-attention mechanisms to capture global context and local geometric information.

Popular architectures include Point Cloud Transformer (PCT) [30], which applies self-attention to point cloud features, allowing the model to focus on the most relevant parts of the input and Superpoint Transformer [31], which hierarchically groups points into superpoints and applies transformers at multiple scales, enabling efficient processing of large point clouds.

Additionally, transformers designed for images, such as SegFormer[32], can be adapted to work with voxel data in a manner similar to 3D CNNs, leveraging self-attention for enhanced 3D performance.

All the above-mentioned architectures have been employed for precision forestry tasks, with an addition of several novel architectures that have been specifically designed for forestry problems.

### Aim of the Review

In this review we aimed to map the landscape of AI methods used in combination with ground-based LiDAR for forestry applications. Specifically, our goal was to address the following questions:

1. What forestry tasks have been solved using AI on ground-based LiDAR data?
2. Which AI models have been used, and which ones show the best results on each task?
3. What data preprocessing methods have been used, and which ones have led to improved results?
4. What are the practices regarding sharing code, sharing data, and ensuring research reproducibility within the field?
5. What AI methods have been successfully applied in other fields and could be used for forestry?

## Methodology

To identify the scientific works relevant for our research questions, we performed a systematic literature review, based on the following selection criteria:

- **Ground-based Data**: Studies were only included if they utilized ground-based LiDAR data. This encompassed TLS and various kinds of MLS, including handheld and backpack-mounted devices. We excluded all airborne laser scanning, which encompasses UAV, satellite and airplane mounted LiDAR.
- **No camera-based point clouds**: We excluded camera-based point clouds obtained with Structure-from-motion and photogrammetry. Such point clouds are dependent on camera settings, environmental conditions and processing methods and are generally considered less robust than laser scanning [33]. To make the comparison between different AI methods as clear as possible we decided to focus on LiDAR data only.
- Use of Machine Learning or Deep Learning: A key criterion was the application of ML or DL techniques for data processing.
- **Individual **Tree** Level Analysis**: The studies needed to address plot level data and provide results at individual tree level. Studies that only provided plot level attributes (e.g. total plot biomass) were excluded.
- **Specificity to **Forest** Data**: The scope was confined to studies involving forest data, excluding research on felled or processed timber, urban trees, and orchards.

Based on these criteria, we have developed the following query:

("forestry" OR "forest" OR "tree") AND ("learning" OR "neural network" OR "artificial intelligence") AND ("LiDAR" OR "point cloud").

The query was applied to the Web of Science database, which yielded 396 results. Titles and abstracts of these studies were manually screened to assess their relevance based on the inclusion criteria. This led to the selection of 39 studies. We identified 13 additional studies that were included based on expert knowledge, resulting in 52 studies in total taken into account.

Following the selection of relevant studies, we conducted a comprehensive analysis of each paper. This process involved categorizing the studies based on the specific forestry tasks addressed and identifying the AI methods employed. We classified these methods as either deep learning or traditional machine learning approaches and we noted the data sources utilized (static TLS, mobile MLS). We also identified the data representations used as input for the AI models, identifying preprocessing operations such as feature extraction, projection and voxelization of the point clouds. For each task category, we identified common performance metrics and collected the reported results.

We also categorized each paper in terms of data and code availability. Code availability was evaluated using simple binary classification, denoting whether the paper had any associated codebase. For data availability, we grouped the papers into three categories:

- Public data: the authors evaluate datasets that have already been published or they collect their own data, which they share publicly along with the paper.
- Own data, shared upon request: the authors collect their own data, which can be accessed by contacting the authors.
- Own data, not available: the authors collect their own data and do not make it available in any way. Some of the works explicitly state that the data cannot be shared, but the majority do not include a data availability statement.

After extracting this information, we evaluated the performance of the different AI methods. Due to vast differences between datasets, we focused on papers that compare different methods on the same data. For each task, our analysis included a general comparison between ML and DL, comparison between specific ML algorithms and DL models, and the impact of different preprocessing methods.

## Results and Discussion

### General view

**Fig. 3:**
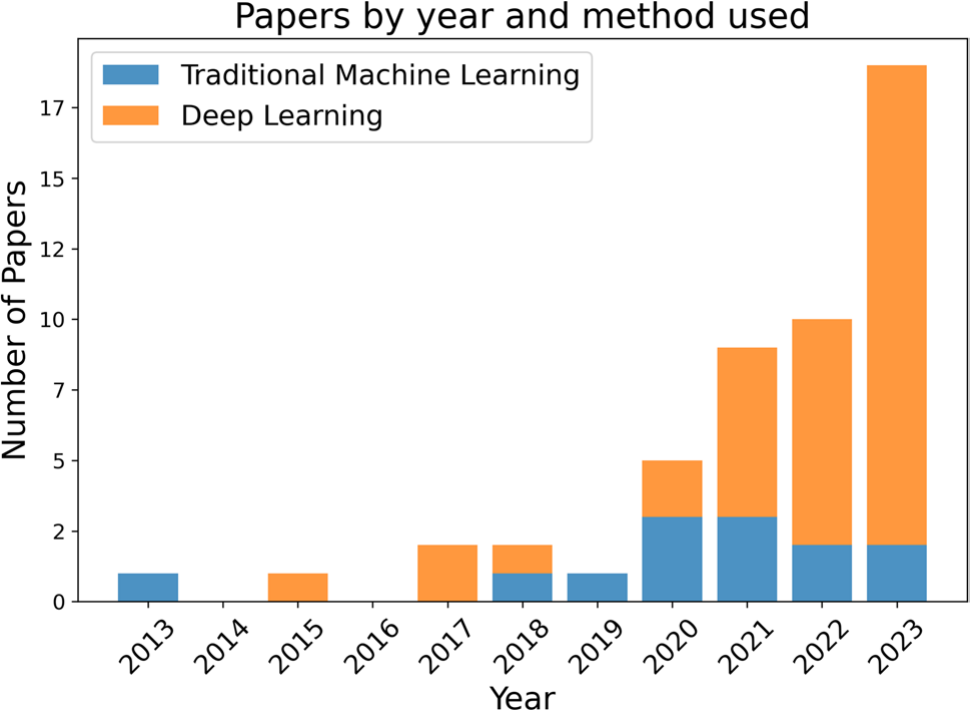
The number of papers making use of Traditional Machine Learning and Deep Learning over time. Several studies employed both types of methods, in such a case we classified it based on the best performing one, which in all such cases was Deep Learning

Starting in 2013, the research landscape on this topic was relatively quiet, with annual outputs ranging from none to a maximum of two papers per year up until 2019, as can be seen in Fig. 3. This pattern shifted in 2020, when the number of publications began to climb noticeably and consistently. The growth trajectory peaked in 2023, which saw an impressive total of 24 papers, with this upsurge largely fueled by DL techniques. In our comprehensive review, 21 studies were found to employ traditional ML algorithms, while 38 studies utilized DL. Notably, seven papers conducted comparative analyses between DL and traditional ML approaches, with DL emerging as the superior performer in all cases.

Our analysis identified that the vast majority of the papers fall into one of three main categories:

- Semantic segmentation (20 studies): This category includes studies focused on delineating various parts of trees, such as leaves, branches, and stems and differentiating other forest elements, such as the ground, shrubs, and woody debris.
- Individual tree segmentation (8 studies): These studies aim to identify separate trees within a point cloud and assign points to the corresponding trees.
- Species classification (15 studies): This task involves providing a species label to a point cloud of a single tree.
- Other tasks (9 studies): This group comprises various studies that don’t align with the above categories.

**Fig. 4:**
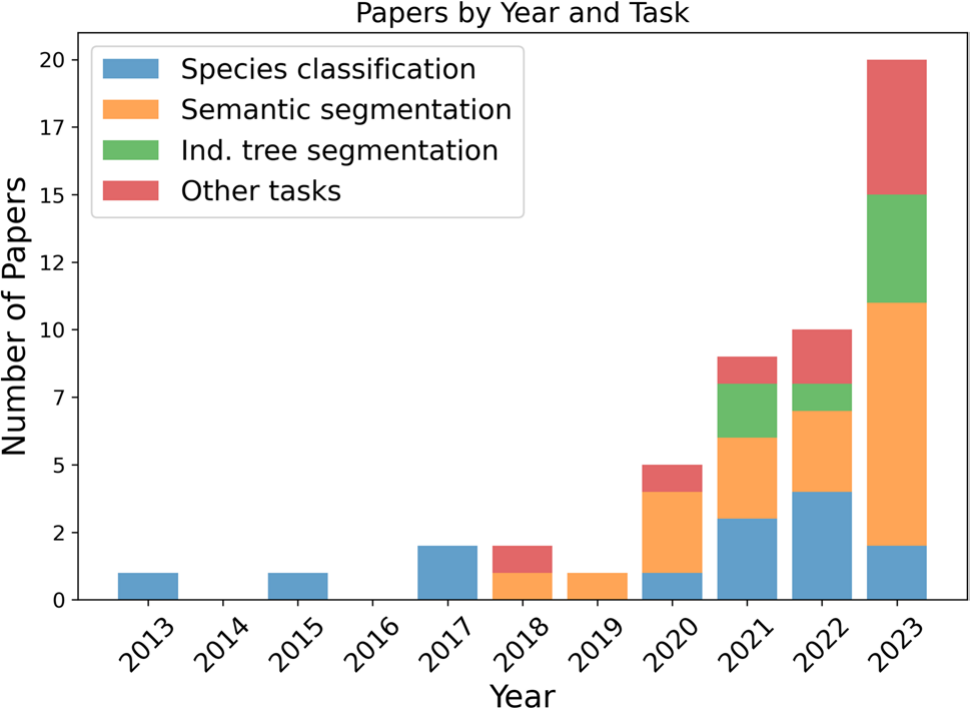
Variation in the number of papers over time focused on different segmentation tasks

Figure 4 outlines how the use of AI in this domain initially centered on species classification, beginning with Othmani et al.’s pioneering study in 2013 [34]. The scope of research broadened to include semantic segmentation by 2018 and further to individual tree segmentation by 2021. The year 2023 stands out for a marked rise in studies focusing on semantic segmentation, which make up almost half of all papers from that year.

**Fig. 5:**
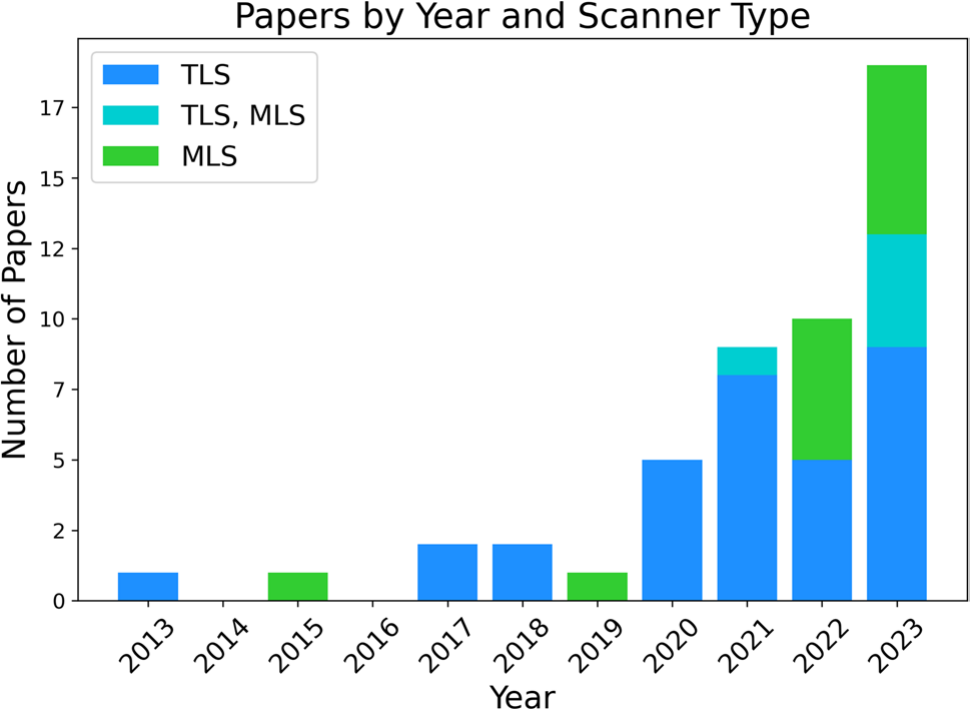
Variation in the number of papers over time that used data from Terrestrial (TLS) and Mobile Laser Scanners (MLS)

Regarding the types of data utilized, shown in Fig. 5, TLS data was the choice for 37 studies, whereas MLS data was used in 18 studies. A total of 5 studies included both scanner types. While TLS was the initially preferred data source, the adoption of MLS rapidly increased, bringing its usage nearly on par with TLS by 2022.

### Point cloud semantic segmentation

This problem involves providing a label to each point based on a predefined list of classes, which might include elements such as stem, branch, leaves, ground, or understory vegetation. The process provides information about the forest structure and is often utilized in other tasks. There are multiple ways to define the classes, depending on the needs of the user. Studies range from basic binary classifications (e.g., wood/non-wood) [8] to more complex systems, such as separating tree points into leaves, stems and branches [35] or categorizing ground components such as shrubs and grass [36]. We summarize each paper approaching this task in Table 2. The papers utilize 7 different evaluation metrics: Overall Accuracy (OA), mean Intersection over Union (mIoU), Precision, Recall, F1, Kappa, Matthews Correlation Coefficient (MCC). In the table, we report the 3 most commonly used metrics: OA, mIOU and F1 score.

**Table 2:**
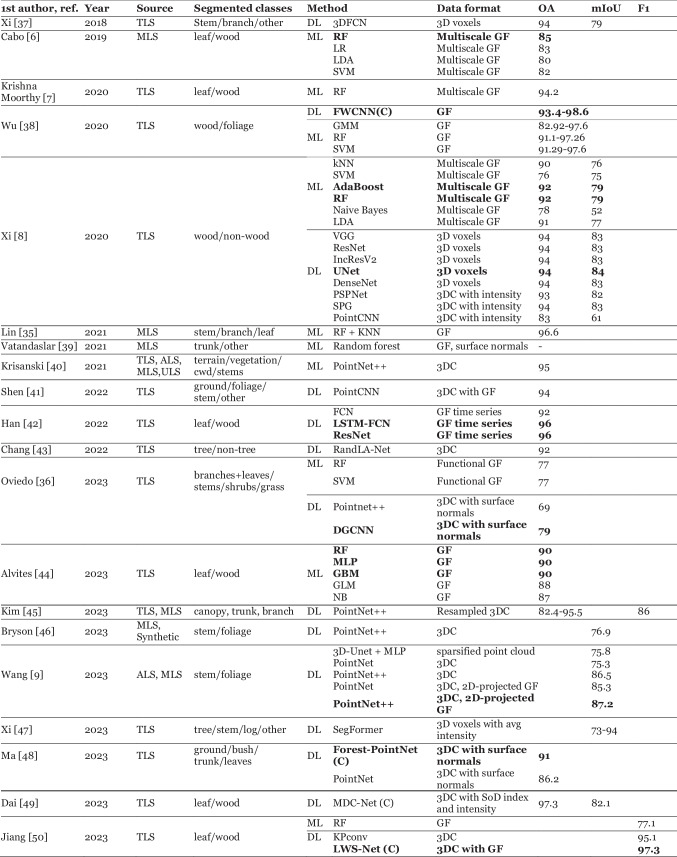
Summary of reviewed papers that address point cloud semantic segmentation. Methods in **bold** indicate the highest performance in their respective studies. Abbreviations used: GF — geometric features, 3DC — 3D coordinates, (C) — custom deep learning architecture

#### Feature-based machine learning

A popular approach involves training a ML model based on geometric features of each point, as described in Sect. 1.1.1. Several papers extract these features across multiple scales [6–8]. A variation of the method involves a functional approach, proposed by Oviedo et al. [36], in which each geometric feature is represented as a continuous function of the neighbourhood size. The studies evaluated many different ML models. The review suggests that Random Forest performs especially well, being the top method in 4 studies[6, 7, 35, 44]. SVM and XGBoost have shown to perform similarly well, with only marginally poorer performance. In the study by Xi et al. [8] AdaBoost, an ensemble model similar to RF achieved the best result.

### Deep Learning

A majority of semantic segmentation papers involve DL. A particularly popular model is PointNet +  + , with 5 papers utilizing it [9, 36, 40, 45, 46]. Works from Wang et al. [9] and Ma et al. [45] showed that additional information in form of geometric features resulted in better performance. Wang et al. [9] develop their own feature extraction method, in which point neighbourhoods are projected on 2D planes and used to create histograms.

Several works employed other established DL architectures, such as PointCNN [41], LSTM [42] or SegFormer [47], combined with additional processing steps. Xi et al. [47] developed a hierarchical segmentation process, with major classes extracted using SegFormer, and further divided using hand-crafted procedures. Another method from Han et al. [42] described points as sequences of geometric features of their neighbours, ordered by distance. This enabled the use of a sequence-based DL model, LSTM-FCN. Shen et al. [41] employed PointCNN combined with a technique which involves adjusting point locations based on geometric features.

Studies by Wu et al. [38], Jiang et al. [50] and Dai et al. [49] have ventured into designing their own custom DL architectures. All three propose models that make use of geometric features of points, and [38, 49] and additionally take into account intensity information. All the studies show improved results compared to methods that only use 3D point coordinates.

#### Benchmarking

A benchmarking study by Xi et al. [8] involved evaluating different semantic segmentation methods, including six traditional ML methods based on geometric features, six voxel-based DL models, and two point cloud DL methods. The DL models, both voxel and point-based, showed similar performance and outperformed traditional ML classifiers by about 10% on average, showing the general effectiveness of DL regardless of specific architecture.

#### Key takeaways

• DL models generally outperform traditional ML models.

• Geometric and intensity features provided alongside 3D coordinates improve the performance of DL models.

• PointNet +  + is a popular and reliable model choice, but there is no clearly superior DL architecture.

### Individual tree segmentation

A fundamental precision forestry task is separating individual trees from a point cloud. It can be framed as instance segmentation, a Computer Vision problem in which each point is assigned a label denoting the specific object it belongs to. We summarize the papers in the area in Table 3. The papers utilize 7 different evaluation metrics (F1, Accuracy, Detection rate, mIoU, AP, Recall, Precision). The two most commonly used metrics are F1 and detection accuracy.

**Table 3:** Summary of papers dealing with individual tree segmentation

1st Author, ref	Year	Data source	Method		Data Representation	F1	Accuracy
Hui [51]	2021	TLS	ML	PCA, GMM	Point cloud	-	69.8
Xi [52]	2021	TLS	DL	CenterNet	2D voxels	75.4	-
Chang [43]	2022	TLS	DL	YOLOv3 + hierarchical clustering	2D feature maps	89.4–94.1	-
Zhou [53]	2023	MLS	DL	Improved PointPillar	Vertical columns of points	85	52.5
Henrich [54]	2023	MLS, TLS	DL	TreeLearn (custom)	3D voxels	98.2	-
Zhang [55]	2023	MLS	DL	WCF-CACL-RandLA-Net (custom)	Point cloud	-	69.9
Xiang [56]	2023	MLS, UAV-LS	DL	Custom	Point cloud	68.9	-
Wielgosz [57]	2024	TLS, MLS, UAV-LS	DL	Custom (from Xiang)	Point cloud	84.5	-

There exist a variety of non-learning approaches for the task. Many of them rely on detecting tree stems, which are usually clearly separated and easy to identify. The remaining points are assigned to the stems based on rules and heuristics, using tools such as density-based clustering, graph connectivity or geometric features [54]. While these approaches show reasonable performance, especially in evenly planted artificial forests, they tend to fail in dense forests with intersecting crowns and subcanopy trees. Such rule-based approaches are rather inflexible and often require extensive manual finetuning.

#### Hybrid approaches

Several papers propose hybrid approaches, applying AI models for parts of the pipeline and integrating them with rule-based methods. In works by Hui et al. [51] and Zhang et al. [55] an AI model is used only for semantic segmentation of trunk points, which are then used for clustering.

Another approach, employed by Xi et al. [52] and Zhou et al. [53] involves locating trees with rectangular bounding boxes. This method is relatively simple to implement and train compared to methods that label each point separately, but rectangular boxes do not capture the exact tree shape and are prone to error when dealing with overlapping crowns. Therefore, works embracing this framework rely on additional processing steps to label individual points.

#### Instance segmentation with offset prediction

Individual tree segmentation can be framed as an offset prediction problem. In this approach, the model learns to map each point to a corresponding tree base using an offset vector. When the points are moved using these vectors, points from a single tree form well-separated clusters. This framing turns the discrete classification problem into continuous regression, which makes the model training easier. Henrich et al. [54] employ this approach in TreeLearn, a UNet-based model for offset prediction. A similar approach was proposed by Xiang et al. [56]. The method was trained on both urban and forest datasets and can segment trees, as well as other objects such as cars or buildings. The same architecture was employed by Wielgosz et al. [57], who used downsampling to generate point clouds of various density, down to the resolution of 10 points/m^2^, typical for aerial data. They showed that the downsampling was beneficial for the training, with a single model performing well on a wide range of densities. In addition to being versatile, the model showed improved performance even on the original, high-density data.

#### Potential DL approaches

While these approaches show promising results, there are many other instance segmentation models that have not been applied for individual tree segmentation. Architectures such as Mask3D [58] or SPFormer [59] have shown remarkable performance on indoor datasets and they are worth investigating in the context of forests. Other interesting works include OneFormer3D [60], which performs semantic and instance segmentation with one model, and FreePoint [61], which learns instance segmentation in an unsupervised manner.

#### Key takeaways

• Heuristic rule-based approaches remain popular, machine learning approaches are relatively less developed.

• Many methods utilize DL models combined with clustering and postprocessing techniques.

• A promising research direction involves DL models for offset prediction.

• Many DL models for instance segmentation (e.g. SPFormer, Mask3D) still have not been tried for individual tree segmentation.

### Species classification

This task involves identifying the tree species based on a point cloud of a single tree. The papers dealing with this problem are summarized in Table 4.

**Table 4:**
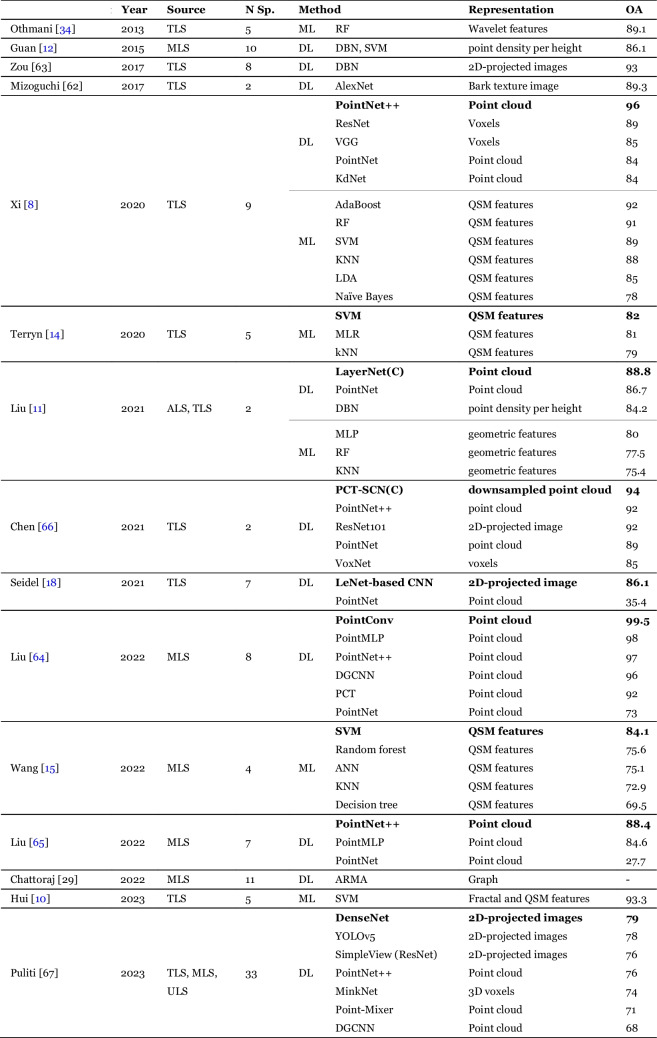
Different approaches for species classification. Abbreviations: OA — Overall Accuracy, (C) — custom deep learning architecture, N Sp. — Number of tree species

#### Early work: bark classification

Species classification has been tackled with machine learning already in 2013 by Othmani et al. [34] and Mizoguchi et al. in 2017 [62]. Both approached the task by modelling the texture of the tree bark. This approach has since been abandoned with later methods instead relying on the shape of the whole tree.

#### Traditional machine learning

Guan et al. [12] characterized trees by counting points at various heights and constructing a vertical distribution. The distribution was further transformed with a Deep Boltzmann machine and classified with an SVM.

A popular class of approaches involves ML based on features extracted from a QSM model [8, 10, 14, 15], which provides a comprehensive description of the branching structure. A study by Hui et al. [10] showed that combining QSM with directly measured and fractal features led to improved results.

#### Deep learning

Several studies investigated projecting point clouds as 2D images and applying image-based DL models to predict the species. Seidel et al. [18] showed that a simple CNN based on a single image was able to significantly outperform a PointNet model. Zou et al. [63] utilized 36 images, rotated around the vertical axis, while Allen et al. [19] used 6 depth-colored images. Another line of work involves point-based DL. Two studies by Liu et al. [64, 65] compared different DL architectures, with PointConv and PointNet +  + being the top performing models. All architectures performed similarly, except for PointNet which was consistently worse. Several works showed that the DL performance benefitted from point cloud downsampling, specifically by using farthest point sampling to a fixed number of points (1024 points in [66] and 2048 point in [64, 65]). Xi et al. [8] compared three classes of methods: QSM-based ML, Voxel DL and Point cloud DL. The best result was achieved by PointNet +  + , followed closely by two QSM-based models: AdaBoost and RF. Another benchmarking study, in a form of a data science competition was performed by Puliti et al. [67] and it involved 7 different DL methods. The three top-performing methods were CNN models on 2D images, with PointNet +  + being a strong contender.

A unique study by Chattoraj et al. [29] reformulated the species classification problem as multi-classification of tree structural attributes: phyllotaxis, divergence and branching mode, which enabled identification of 11 species. In addition, they represented the point cloud as a graph and classified it using a Graph Neural Network.

#### Key takeaways

• QSM provides good features for species classification.

• Projecting a point cloud onto 4–6 images and processing them with a CNN works remarkably well.

• Point-based DL works well, PointNet +  + shows consistent strong performance.

 For point cloud DL, farthest point downsampling to a fixed number of points improves performance.

• PointNet does not work well, should be avoided.

### Other tasks

While most of the studies focus on the tasks described above, they are not the only ones. The tree can be characterized in more detail by providing a model of the branching structure, detecting tree damage or identifying microhabitats present on the tree. The papers not fitting into previous categories are summarized in Table 5. Since the papers deal with diverse, often novel problems, without clearly established evaluation metrics, we do not provide numerical results here.

**Table 5:**
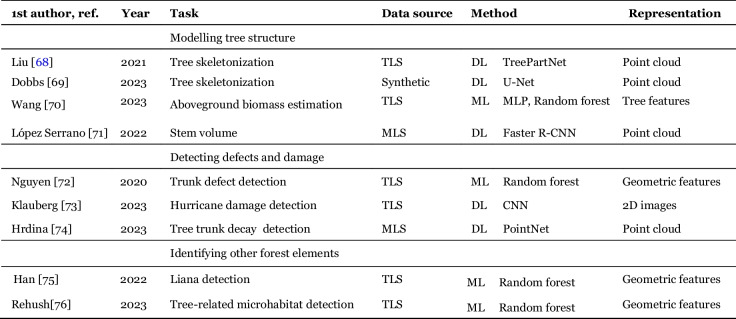
Papers approaching various tasks that do not fit into the previous categories

#### Modelling tree structure

Several works have been dedicated to model the structure of the tree and its parameters. Wang et al. [70] use TLS-derived features to predict aboveground biomass of individual trees. Lopez Serrano et al. [72] use a DL model to predict stem volume, showing improved performance over handcrafted methods. More detailed representation of tree structure can be done with skeletonization, i.e. converting a point cloud into a model of the branching structure. Methods by Dobbs et al. [69] and Liu et al. [68] present DL-based approaches that provide an alternative to handcrafted QSM algorithms.

#### Detecting defects and damage

An important application involves detecting various kinds of tree damage. Klauberg et al. [73] used TLS data to assess post-hurricane damage at individual tree level. They developed a projection-based method, involving 12 viewing angles around the vertical axis, classified with CNN models. They found VGG16 to be the best performing architecture. Hrdina et al. [74] developed a method for internal trunk decay detection. They collected MLS data and ground-truth based on acoustic tomography and developed a PointNet-based classifier. Nguyen et al. [72] developed a ML approach for classifying tree trunk defects. They used a handcrafted clustering method for detecting defective segments, and a RF classifier to determine the type of defect.

#### Identifying other forest elements

The detail present in ground-based LiDAR makes it possible to identify various non-tree parts of forest ecosystems. Han et al. [75] developed a model for detecting lianas growing on trees using geometric features and RF. Rehush et al. [76] used TLS data to detect 6 classes of tree-related microhabitats (Bark, Bark pockets, Cavities, Fungi, Ivy, Mosses). The authors framed the task as semantic segmentation and tried two different approaches: RF based on geometric features and CNN based on 2D images. They found that the CNN significantly outperformed the RF.

#### Key takeaways

• AI can be used for characterization of individual trees in terms of damage, diseases or branching structure.

• Non-tree elements of forest ecosystems can be detected using AI.

• These areas are relatively undeveloped and open for future research.

### Reproducibility analysis

We investigated three aspects of research reproducibility: availability of code, availability of data, and performance comparison between studies.

#### Code sharing

**Fig. 6:**
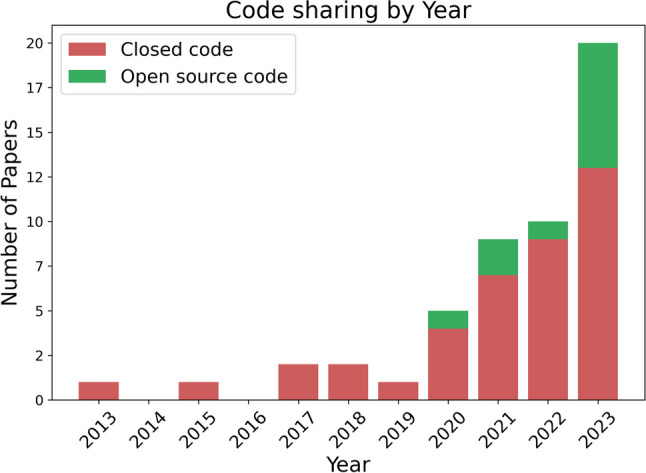
Number of papers sharing their code over the years

Despite a notable increase in code sharing in 2023, as depicted in Fig. 6, the majority of studies, particularly those introducing novel deep learning (DL) architectures [11, 38, 42, 48, 50, 55, 66], still lack accompanying code repositories. This absence hinders the reproducibility of these complex models, which cannot be effectively reconstructed from text descriptions alone. As a result, the impact and credibility of such research are significantly reduced. More robust code sharing practices could greatly enhance the scientific community’s ability to verify and build upon new findings.

#### Data sharing

**Fig. 7:**
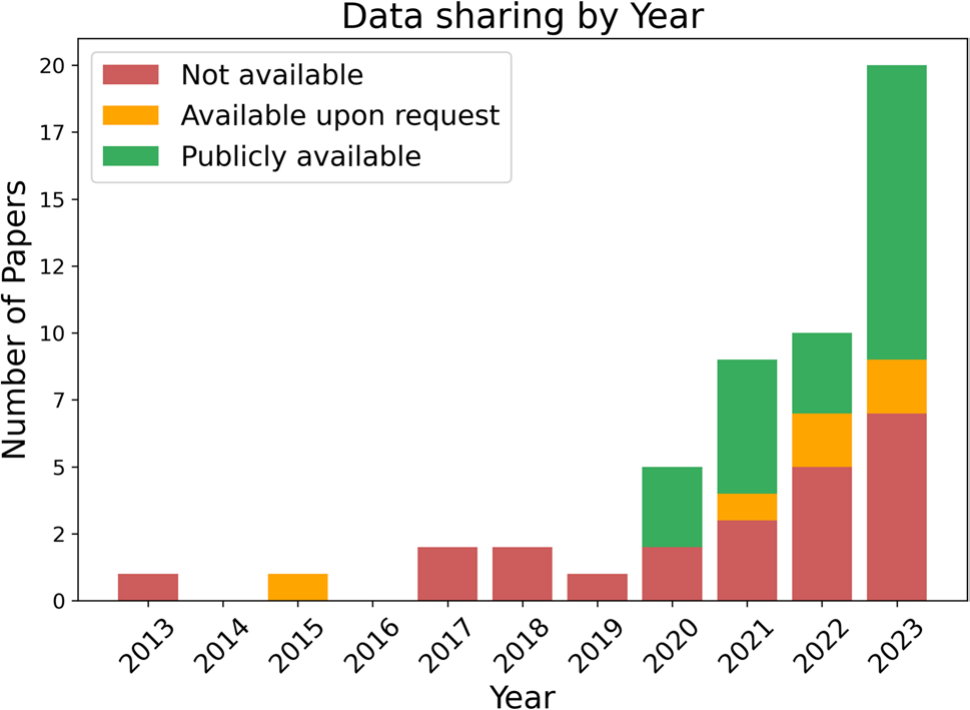
Number of papers utilizing publicly available data over the years. The three studies in 2023[9, 44, 68] that used both public and closed datasets were classified as using public data

Figure 7 shows that 44.2% of the reviewed papers evaluate their methods on self-collected datasets, and do not provide access to them. This practice was especially prevalent in the early years. There is a clear upward trend in data sharing over time, especially significant in 2023. However, there is still a significant number of new papers that collect their own data and do not make it available. Utilizing public datasets makes it easier to compare and benchmark different methods and is a sign of the field becoming more structured and mature.

Several papers [11, 38, 42, 48, 50, 55, 66] follow a pattern which makes them practically impossible to reproduce or evaluate. They propose a novel DL architecture for a specific forestry task. While they describe the network architecture, they do not provide open-source code. In addition, they only evaluate their method on a self-collected dataset with no open access. This way, they make it impossible to test their approach on other datasets, or to test different approaches on their data.

#### Benchmark datasets

While the majority of papers use their own data, 37.3% of works make use of publicly available data from other sources. Several datasets have been used in multiple papers:

- **ISPRS/EuroSDR benchmark** [77]: a dataset launched in 2014, with 24 sample plots encompassing diverse species, growth stages, and management conditions in a southern boreal forest in Finland. It contains high-density TLS data with both single-scan and multiscan point clouds. The dataset contains reference data including positions of individual trees, making it a valuable resource to evaluate individual tree detection methods. The data has been used by 4 studies in this review [8, 43, 51, 52].
- **Wytham woods** [78]: a dataset made available in 2016, containing data about 835 individual trees, including segmented point clouds and QSM models. Used by 2 studies: [14, 57]
- **FOR-species** [67]: a combined dataset from several countries, containing approximately 20,000 individual tree point clouds with species labels. It was introduced for a benchmarking project with 7 submissions proposing different tree species classification approaches.

#### Evaluation metrics

A problem we encountered while performing this review involved inconsistent performance metrics. We found 7 metrics for semantic segmentation: OA, mIoU Precision, Recall, F1, Kappa, MCC; 7 metrics for individual tree segmentation: F1, Accuracy, Detection rate, mIoU, AP, Recall, Precision; and 5 metrics for species classification: OA, Precision, Recall, F1-score, Kappa. The only metric that was used consistently within a group was OA for species classification. For other tasks, none of the metrics were used consistently. While it is important to use a variety of metrics to capture different aspects of the problem, there is a need for clearly defined standardized metrics used across the field.

### Perspective on AI Methods in Forestry

One of the goals of this review was to evaluate to what degree the precision forestry field utilizes the latest AI research, and what areas might be overlooked.

#### Deep learning architectures

In general, a wide range of DL models have been investigated. This includes various point-based DL architectures. The ones that have proven to work well include: PointNet +  + , PointCNN, PointMLP. Architectures such as PCT, DGCNN, KPConv were shown to be slightly worse, perhaps because of limited dataset sizes. PointNet has shown to be consistently inferior and should probably be avoided.

In case of 2D-projected images and 3D voxel grids, it seems that any established computer vision model, such as ResNet or VGG, will do the job well.

Progress is still to be made in architectures for individual tree segmentation. There is relatively little work in the area, and all the promising DL methods are based on offset prediction [56, 57]. They show remarkable results outperforming the rule-based approaches but there is clearly room for development. Research in the area of indoor and urban instance segmentation might be used as a source of inspiration.

A large area that is almost entirely unexplored is the use of Graph Neural Networks (GNNs). This popular and diverse family of models is almost completely absent in the reviewed articles, apart from [29]. The branching structure of trees has an inherent graph nature, which can be represented in form of QSM or other graph formats. Perhaps such a representation might provide important abstractions that are not easily extracted from raw point clouds. In addition, the whole forest can be modeled as a graph of connected trees. For tasks such as species classification, it might be beneficial to consider the tree not as an isolated point cloud, but as a part of a tree network, which largely constrains the composition of species in a given area.

#### Data preprocessing

Choosing the right methods for data preprocessing can have a significant impact on the performance of AI models. One essential technique is data augmentation, which involves applying various disturbances to the training data to generate more samples and improve the model's generalization capabilities. Data augmentation has been employed in 15 of the reviewed papers. The most common technique was point cloud rotation [8, 9, 18, 19, 40, 46–48, 53, 55, 56], typically along the vertical axis, though some studies [40] incorporate small rotations along other axes, resulting in slight tilts of the trees. Other prevalent methods include jittering [40, 56, 74], which involves adding Gaussian noise to point coordinates; scaling [19, 53, 55, 56], downsampling [46, 57] and mirror-flipping [52, 53, 56, 76] of the point cloud. Studies that explicitly evaluated the impact of data augmentation [8, 18, 57, 76] consistently reported improved model performance when trained on augmented data.

Another practice that has shown good results, specifically in semantic segmentation, is enhancing point representations with additional handcrafted features. Multiple studies [9, 44–46] have shown that providing geometric and intensity features can support the performance of DL models.

#### Expanding the datasets

While exploring various types of model architectures is important, a huge potential for improvement lies in expanding the datasets. In line with a critical analysis by Lines et al. [79], we think that the development of large-scale, international benchmark datasets is crucial to move the field forward. To facilitate the development of such datasets, it is essential to establish comprehensive data standards. These standards should encompass clearly defined data formats, label structures, and evaluation methods to ease the process of combining data from multiple sources and coordinated evaluation of different models.

A promising example of this standardization is already emerging in the domain of individual tree segmentation, initiated by Puliti et al. creating the FOR-instance dataset [79] of labeled UAV-LS scans from five different countries. Their approach introduces a consistent data format using.laz files with an additional treeID field for each point, coupled with a clear evaluation guideline and a predefined train-test split. Building on this work, Henrich et al. [80] have processed two existing ground-based datasets (Wytham Woods and LAUTx) to fit this standard, further promoting its adoption and contributing to the data pool.

It might be beneficial to combine data from various LiDAR modalities, both ground-based and aerial. Krisanski et al. [37] and Wielgosz et al. [54] show that it is possible to train sensor-agnostic models, which might even outperform their modality-specific counterparts. This approach makes it easier to create large datasets and it provides models with wider applicability.

This can be supported by synthetic data. There is a vast computer graphics literature focused on developing realistic tree-growth simulations. Such simulations can be used to quickly generate large amounts of samples, with labels automatically provided. Several studies in this review [43, 66] showed that employing synthetic data has led to improved performance in real-world evaluation. While synthetic data cannot capture all aspects of a real forest, combining real and synthetic data can certainly be a part of the solution.

#### Alternatives to supervised learning

An area of AI research largely overlooked in the reviewed articles includes learning paradigms other than pure supervised learning, specifically semi-supervised and self-supervised learning.

Semi-supervised learning involves training a model using a mix of labeled and unlabeled examples. A popular technique, self-training, involves the model teaching itself by incorporating its own high-confidence predictions into the learning process. Another approach might include training based on predictions from an ensemble of different models. The semi-supervised paradigm was only employed in one reviewed paper [9], which showed improved performance through self-training.

Another overlooked paradigm is self-supervised learning, in which a model can learn useful features from large unlabeled datasets. This approach has been very successful in 2D computer vision [82] and natural language processing [83]. Self-supervised learning leverages inherent structural properties of data to create supervisory signals. By learning to predict hidden or transformed parts of the input, models develop rich internal representations applicable to downstream tasks. Various methods of self-supervision have been developed for point cloud data. This includes shape completion, in which a small region of the point cloud is masked and a model is trained to predict the positions of the points within it. It is typically implemented using a masked autoencoder architecture, with popular ones being Point-MAE [84] and Voxel MAE [85]. Another approach is point upsampling, in which the model learns by trying to increase the density of downsampled point clouds. An example of such approach is SAPCU [86].

An increasingly popular paradigm in the AI community is generative modelling. Generative models, such as Generative Adversarial Networks (GANs), variational autoencoders, or diffusion models, have demonstrated remarkable capabilities in synthesizing various kinds of realistic outputs, including tree structures and growth patterns, as seen in works like DeepTree [87]. These models can be harnessed not only for generating synthetic datasets that supplement LiDAR data, but also as predictive tools for understanding and simulating forest dynamics. By training generative models on LiDAR data, they could potentially predict future growth patterns and provide insights into forest development under various conditions. Furthermore, the internal representations learned by generative models could be used for feature extraction, potentially leading to more robust tree classification and segmentation. Exploring the interplay between generative models and LiDAR data could reveal new ways to tackle challenges in forest monitoring.

#### Overlooked tasks

Relatively little research has been done to assess attributes of individual trees beyond their species. The detail present in ground-based scans could potentially be used to identify dead and infected trees, and to predict their age class. All this information is crucial for forest management.

Dead tree detection stands out as a particularly promising task that has received little attention. It is likely the easiest of these overlooked tasks to implement, as labeling dead trees in datasets is relatively straightforward and feasible based on the scan itself. Dead tree detection holds high relevance for various aspects of forest ecology and management, including assessing forest health, estimating carbon stocks, and managing fire risks.

Tree disease detection, despite its importance, was addressed in only one paper reviewed, addressing the specific issue of tree trunk decay [74]. AI models could be used to generally identify trees with poor health, and perhaps to specifically identify the most common pathogens. Early and accurate detection of disease spread is crucial for making management decisions.

Another important issue is tree age prediction. It could be implemented either as continuous regression predicting a specific number or age class classification, predicting one of several discrete age groups. However, acquiring accurate ground truth data for tree age is often difficult and labor-intensive. 

While current AI architectures are fully capable of solving these important challenges, the key constraint is the requirement to collect large, diverse datasets with reliable ground-truth labels. Such datasets are critical for developing models that can operate across a wide range of ecological settings.

## Conclusion

This review underscores the pivotal role of Artificial Intelligence, particularly Deep Learning, in transforming precision forestry through the analysis of ground-based LiDAR data. Our comprehensive examination has revealed that the primary applications of AI in this field center around semantic segmentation, individual tree segmentation, and species classification. Among the array of models tested, deep learning architectures like PointNet+ + have consistently outperformed traditional machine learning methods, offering enhanced accuracy and efficiency.

Despite these advancements, the study identifies several underexplored areas such as the potential of Graph Neural Networks and other novel DL models that could provide significant breakthroughs in how we understand and manage forest environments. Moreover, the review highlights a critical gap in the field: the scarcity of shared code and standardized metrics, which complicates the replication of research and limits the comparative analysis of different approaches.

To propel the field forward, we advocate for the establishment of large, comprehensive benchmark datasets that combine data from diverse environments and LiDAR modalities. Such datasets would not only facilitate the development and testing of new models but also ensure that rigorous evaluation standards are met across studies. Additionally, fostering an environment of open scientific communication by making datasets and DL models publicly accessible will be crucial for collaborative advancements in precision forestry.

In conclusion, while AI methodologies have shown considerable promise in enhancing the capabilities of forest monitoring and management, the realization of their full potential will depend heavily on concerted efforts to address current limitations. By embracing open data practices, standardizing evaluation metrics, and exploring innovative AI architectures, the field of precision forestry can advance in accuracy and efficiency, paving the way for detailed forest observation and sustainable management practices.

## Data Availability

No datasets were generated or analysed during the current study.
